# Supplementary material for: Prokaryotic viruses impact functional microorganisms in nutrient removal and carbon cycle in wastewater treatment plants
Source: Nat Commun. 2021 Sep 13;12:5398. doi: 10.1038/s41467-021-25678-1 (PMC8438041; doi:10.1038/s41467-021-25678-1)
Supplement: Supplementary file 2 — Description of supplementary files [file 41467_2021_25678_MOESM2_ESM.pdf]

Description of Additional Supplementary Files

File Name: Supplementary Data 1

Description:

Supplementary Data 1. Diversity of viral genera in 6 WWTPs (Shannon's diversity index H' and Pielou's evenness index J').

File Name: Supplementary Data 2

Description:

Supplementary Data 2. Host prediction for shared viral genera between Sample AS and IMG/VR database (phylum level)

File Name: Supplementary Data 3

Description:

Supplementary Data 3. Host prediction for shared viral genera between Sample AS and IMG/VR database (genus level)

File Name: Supplementary Data 4

Description:

Supplementary Data 4. Characteristics of phage genomes included in the mock communities.

File Name: Supplementary Data 5

Description:

Supplementary Data 5. Nucleotide identity and amino acid identity with reference phage genomes (Mock community dataset)

File Name: Supplementary Data 6

Description:

Supplementary Data 6. Nucleotide identity and amino acid identity with reference phage genomes (Mock community datasets pooled in 6 WWTP samples)

File Name: Supplementary Data 7

Description:

Supplementary Data 7. Parameter optimization for CRISPR-Cas spacer matching

File Name: Supplementary Data 8

Description:

Supplementary Data 8. Number of spacers in each Midas genus genome

File Name: Supplementary Data 9

Description:

Supplementary Data 9. Filtered virus-host connections in Hi-C data (Illumina)

File Name: Supplementary Data 10

Description:

Supplementary Data 10. Virus-host connections predicted with CRISPR spacers in Hi-C data (Illumina)

File Name: Supplementary Data 11

Description:

Supplementary Data 11. Filtered virus-host connections in Hi-C data (Nanopore)

File Name: Supplementary Data 12

Description:

Supplementary Data 12. Virus-host connections predicted with CRISPR spacers in Hi-C data (Nanopore)

File Name: Supplementary Data 13

Description:

Supplementary Data 13. Comparison of 11 detected virus-host interactions for Illumina Hi-C data (91% precision).

File Name: Supplementary Data 14

Description:

Supplementary Data 14. Comparison of 16 detected virus-host interactions for Nanopore Hi-C data (94% precision).
