# Supplementary material for: Prokaryotic viruses impact functional microorganisms in nutrient removal and carbon cycle in wastewater treatment plants
Source: Nat Commun. 2021 Sep 13;12:5398. doi: 10.1038/s41467-021-25678-1 (PMC8438041; doi:10.1038/s41467-021-25678-1)
Supplement: Supplementary file 17 — Reporting Summary [file 41467_2021_25678_MOESM17_ESM.pdf]

nature portfolio

Corresponding author(s): TONG ZHANG

Last updated by author(s): Aug 12, 2021

Reporting Summary

Nature Portfolio wishes to improve the reproducibility of the work that we publish. This form provides structure for consistency and transparency in reporting. For further information on Nature Portfolio policies, see our Editorial Policies and the Editorial Policy Checklist.

Statistics

For all statistical analyses, confirm that the following items are present in the figure legend, table legend, main text, or Methods section.

n/a	Confirmed	
☐	☒	The exact sample size (n) for each experimental group/condition, given as a discrete number and unit of measurement
☐	☒	A statement on whether measurements were taken from distinct samples or whether the same sample was measured repeatedly
☒	☐	The statistical test(s) used AND whether they are one- or two-sided<br>*Only common tests should be described solely by name; describe more complex techniques in the Methods section.*
☒	☐	A description of all covariates tested
☒	☐	A description of any assumptions or corrections, such as tests of normality and adjustment for multiple comparisons
☒	☐	A full description of the statistical parameters including central tendency (e.g. means) or other basic estimates (e.g. regression coefficient) AND variation (e.g. standard deviation) or associated estimates of uncertainty (e.g. confidence intervals)
☒	☐	For null hypothesis testing, the test statistic (e.g. F, t, r) with confidence intervals, effect sizes, degrees of freedom and P value noted<br>*Give P values as exact values whenever suitable.*
☒	☐	For Bayesian analysis, information on the choice of priors and Markov chain Monte Carlo settings
☒	☐	For hierarchical and complex designs, identification of the appropriate level for tests and full reporting of outcomes
☒	☐	Estimates of effect sizes (e.g. Cohen's d, Pearson's r), indicating how they were calculated

Our web collection on statistics for biologists contains articles on many of the points above.

Software and code

Policy information about availability of computer code

Data collection	No software was used.
Data analysis	CLC Genomics Workbench (version 11.0.1) ,Prodigal V2.6.3, HMMER 3.1b2, DIAMOND v0.9.25, MEGAN6, Nucmer 4.0.0beta2, CompareM v0.0.23, vContact2 (version 0.9.5), ClusterONE-1.0, Cytoscape (version 3.7.1), CRT 1.2, BLAST 2.9.0+, emapper-1.0.3-40-g41a8498, CD-HIT 4.8.1, OPERA-MS (v0.8.3), https://github.com/yqchen17/Manuscript

For manuscripts utilizing custom algorithms or software that are central to the research but not yet described in published literature, software must be made available to editors and reviewers. We strongly encourage code deposition in a community repository (e.g. GitHub). See the Nature Portfolio guidelines for submitting code & software for further information.

Data

Policy information about availability of data

All manuscripts must include a data availability statement. This statement should provide the following information, where applicable:

- Accession codes, unique identifiers, or web links for publicly available datasets
- A description of any restrictions on data availability
- For clinical datasets or third party data, please ensure that the statement adheres to our policy

All raw sequencing data from the six AS viromes generated in this study have been deposited in the NCBI Sequence Read Archive (SRA) database under BioProject ID: PRJNA639411 (https://www.ncbi.nlm.nih.gov/Traces/study/?acc=PRJNA639411). Hi-C raw sequencing data generated in this study have been deposited in the NCBI Sequence Read Archive (SRA) database under BioProject ID: PRJNA745436 (https://www.ncbi.nlm.nih.gov/Traces/study/?acc=PRJNA745436).
All complete viral genomes of whose hosts are bacteria and archaea (N=2309) used in this study are available in the NCBI Refseq database V91 (https://ftp.ncbi.nlm.nih.gov/refseq/release/release-catalog/archive/RefSeq-release91.catalog.gz). All bacterial and archaeal assembled genomes (N=190,078) used in this

study are available in the NCBI Assembly database (https://www.ncbi.nlm.nih.gov/assembly). Viral contigs from other ecosystems used in this study are available in the IMG/VR database (https://genome.jgi.doe.gov/portal/IMG_VR/IMG_VR.home.html).
Mock community virome Illumina sequencing reads used in this study are available in http://mirrors.iplantcollaborative.org/browse/iplant/home/shared/iVirus/DNA_Viromes_library_comparison. Source data are provided with this paper.

Field-specific reporting

Please select the one below that is the best fit for your research. If you are not sure, read the appropriate sections before making your selection.

☐ Life sciences ☐ Behavioural & social sciences ☒ Ecological, evolutionary & environmental sciences

For a reference copy of the document with all sections, see nature.com/documents/nr-reporting-summary-flat.pdf

Ecological, evolutionary & environmental sciences study design

All studies must disclose on these points even when the disclosure is negative.

Study description	We amassed a catalog of ~50,000 prokaryotic viruses from six wastewater treatment plants (WWTPs) in Hong Kong.
Research sample	Rationale: We have sampled all six secondary WWTPs in Hong Kong that applied biological treatment methods to remove pollutants. We chose activated sludge (AS) samples since AS is the main process for nutrient removal and carbon cycle in WWTPs. By enriching viruses in the samples, we meant to represent prokaryotic viruses in AS samples.
Sampling strategy	We took grab samples (2L) from the aeration tank of WWTPs. Our preliminary tests showed that 2L was sufficient for DNA extraction.
Data collection	Y.C used the desktop computer to download DNA sequencing data from the Aliyun link offered by Novogene (China).
Timing and spatial scale	Sha Tin (22.418N, 114.215E), Stanley (22.225N, 114.211E), Shek Wu Hui (22.521N, 114.119E), Tai Po (22.471N, 114.187E), and Yuen Long (22.484N, 114.026E) were sampled on 2018.12.05 and Sai Kung (22.388N, 114.270E) sampled on 2018.09.05. We selected these WWTPs since they all applied activated sludge treatment process to remove pollutants. They all located in Hong Kong.
Data exclusions	No data were excluded from the analyses.
Reproducibility	We did an additional sampling in 2020.12 from Sha Tin WWTP to validate the virus-host connections predicted by our CRISPR-based methods. Results show that CRISPR-based results have a very high accuracy (>90% precision).
Randomization	Randomization is not applicable to this because the reported study is a survey of AS viromes in Hong Kong.
Blinding	Not applicable. Because AS viromes are dark matter, we don't have expected results of the composition, diversity, host information and shared viral genera in our samples.

Did the study involve field work? ☐ Yes ☒ No

Reporting for specific materials, systems and methods

We require information from authors about some types of materials, experimental systems and methods used in many studies. Here, indicate whether each material, system or method listed is relevant to your study. If you are not sure if a list item applies to your research, read the appropriate section before selecting a response.

Materials & experimental systems

n/a	Involved in the study	
☒	☐	Antibodies
☒	☐	Eukaryotic cell lines
☒	☐	Palaeontology and archaeology
☒	☐	Animals and other organisms
☒	☐	Human research participants
☒	☐	Clinical data
☒	☐	Dual use research of concern

Methods

n/a	Involved in the study	
☒	☐	ChIP-seq
☒	☐	Flow cytometry
☒	☐	MRI-based neuroimaging
